# Patient-Transition Networks, Multiservice Trajectories, and Specialty Activity in a University Dental Clinic: A Retrospective Ecological Study

**DOI:** 10.3390/healthcare14182938

**Published:** 2026-09-10

**Authors:** Diego Gómez-Costa, Pablo Lastra-Prados, Marta Olmos Valverde, Lorena Camarero Aizpurua

**Affiliations:** Department of Nursery and Stomatology, Faculty of Health Science, Universidad Rey Juan Carlos, 28922 Alcorcón, Spainlorena.camarero@urjc.es (L.C.A.)

**Keywords:** dental services, ecological study, administrative data, patient transitions, PageRank, continuity indicator, reproducibility

## Abstract

Background/Objectives: Routine administrative dental data can quantify specialty activity and reconstruct sequential patient movement when operational definitions and analytical provenance are explicit. To reconcile institutional activity through 30 June 2026, describe the derivation of all treatment records, and reconstruct patient transitions across 18 dental services. Methods: The official institutional report was the source of truth for descriptive totals. Patient-level workbooks were used for deduplication and transition analysis after excluding dates later than 30 June 2026. A treatment record was a coded administrative row; appointments and first visits followed the official report definitions. Directed transitions linked different services in consecutive dated patient stages. PageRank, betweenness and modularity were recalculated from the resulting matrix. Results: The official report comprised 98,685 treatment records represented by 476 treatment codes, 24,327 unique appointments and 1351 first visits. There were 7260 unique anonymized patient codes; 13,026 was the non-additive sum of service-specific patient counts. The cutoff-restricted chronology included 3947 multiservice patients and 16,347 dated stages. The network contained 253 directed service pairs and 8777 transitions (density 0.827). The leading PageRank values were RX (0.202), CIRUGÍA BUCAL (0.149), MEA (0.101), MIA (0.088), and MPSI (0.062). Modularity was low (Q = 0.042). Conclusions: The descriptive results are fully reconciled to the official report, while network estimates are restricted to reproducible individual trajectories from 1 January 2025 to 30 June 2026. The observed network characterizes sequential service use and should not be interpreted as proof of formal referral.

## 1. Introduction

Continuity and network analysis require explicit definitions. The institutional first-visit ratio used here is not the patient-level Bice-Boxerman index [1]. PageRank, betweenness and modularity have distinct mathematical meanings and must be derived from an explicit adjacency matrix [2,3,4]. The reporting strategy followed STROBE principles [5].

### 1.1. University Dental Clinics as Complex Healthcare Systems

University dental clinics combine patient care, clinical education, and research within one organization. Patients may therefore attend several clinical services during a course of care, while student rotations, faculty supervision, curricular requirements, and service-specific schedules also influence when and where care is delivered [6,7,8,9,10,11]. Mapping the observed order of service attendance can help academic dental leaders identify heavily used connections and areas that warrant operational review. However, consecutive attendance does not establish that one clinician formally referred the patient to another service.

Increasing life expectancy, greater retention of natural dentition, and the growing prevalence of chronic oral diseases have progressively transformed dental care into a multidisciplinary process requiring coordinated interventions across diagnostic, surgical, restorative, periodontal, prosthodontic, and preventive disciplines. As treatment complexity increases, successful patient management depends not only on the quality of individual clinical procedures but also on the efficiency with which patients transition between specialties throughout the continuum of care.

Within academic institutions, these organizational demands coexist with educational objectives that require students to achieve competency across multiple clinical disciplines. Rotating clinical assignments, supervision by different faculty members, curricular requirements, and specialty-specific scheduling systems may unintentionally fragment patient pathways, generating additional referrals and potentially compromising continuity of care. Balancing educational needs with patient-centered healthcare delivery therefore represents one of the principal organizational challenges facing university dental hospitals.

Healthcare systems increasingly emphasize integrated care models that promote coordination between professionals, optimize resource utilization, reduce unnecessary delays, and improve patient outcomes. In this context, understanding how patients move through multidisciplinary dental services has become essential for identifying inefficiencies, optimizing organizational performance, and developing evidence-based quality improvement strategies within academic dental institutions.

### 1.2. Patient-Transition Networks and Multidisciplinary Care in Academic Dentistry

Comprehensive oral healthcare often involves diagnostic, surgical, restorative, periodontal, prosthodontic, and preventive services [12,13,14,15]. In this study, a patient transition means that different services appear in consecutive dated stages of an anonymized administrative record. It is not synonymous with a documented clinical referral, because referral orders and reasons for attendance were unavailable.

Within university dental clinics, the observed sequence of services may reflect clinical need, educational requirements, student competency targets, faculty availability, or scheduling arrangements. The resulting patient-transition network therefore describes sequential service use rather than clinical decision-making.

A patient-transition network represents clinical services as nodes and observed consecutive attendances as directed edges. Network measures can summarize which services frequently receive or precede transitions, whether some services lie on many of the shortest paths, and whether the network separates into groups. These measures support descriptive service evaluation, but they do not by themselves identify causes, appropriate pathways, delays, satisfaction, or patient outcomes.

Compared with service totals alone, network analysis shows how services are connected through observed patient sequences. For academic dental leadership, this can identify transition patterns that merit closer examination of scheduling capacity, diagnostic workflows, or multidisciplinary coordination. Any operational intervention would still require clinical validation and outcome data.

Understanding patient-transition patterns is relevant to university dental clinics because organizational arrangements can affect both training and care delivery. Concentration of transitions around a few services may indicate high shared demand, whereas sparse or asymmetric connections may prompt review of scheduling or documentation. These are screening signals for further investigation, not evidence of inefficiency or poor-quality care.

### 1.3. Continuity of Care as an Indicator of Healthcare Quality

Continuity of care is widely recognized as one of the fundamental dimensions of healthcare quality and represents a central component of patient-centered care. Rather than reflecting a single clinical encounter, continuity encompasses the extent to which healthcare services are experienced by patients as coordinated, coherent, and connected over time, regardless of the number of professionals or specialties involved. High levels of continuity have consistently been associated with greater patient satisfaction, improved adherence to treatment, better clinical outcomes, and more efficient utilization of healthcare resources [16,17,18,19].

Three complementary dimensions of continuity have traditionally been described. Informational continuity refers to the effective transfer and use of clinical information throughout successive healthcare encounters. Management continuity reflects the consistency and coordination of therapeutic decisions across providers and treatment episodes. Relational continuity concerns the development of sustained therapeutic relationships between patients and healthcare professionals. Together, these dimensions contribute to maintaining coherent care pathways despite increasing healthcare complexity.

Maintaining continuity within university dental clinics presents unique organizational challenges. Patients frequently receive treatment from undergraduate students, postgraduate residents, specialist trainees, and faculty members distributed across multiple departments during prolonged treatment periods. Clinical rotations, academic calendars, and independent specialty scheduling systems may interrupt patient–provider relationships and increase the number of transitions throughout the course of treatment. Consequently, academic institutions must reconcile educational requirements with the delivery of coordinated longitudinal care.

Although continuity of care has been extensively investigated in primary medical care and hospital settings, evidence within academic dentistry remains scarce. Existing dental studies have predominantly focused on patient satisfaction or access to care, while relatively few have explored continuity from an organizational perspective. Furthermore, little is known about how continuity interacts with transition patterns, multidisciplinary care pathways, or organizational network structure within university dental hospitals. Addressing this knowledge gap may contribute to the development of measurable quality indicators capable of supporting continuous improvement initiatives in academic oral healthcare.

### 1.4. Time-to-Treatment as a Performance Indicator in Academic Dental Care

Timely access to dental treatment is a key determinant of healthcare quality, patient satisfaction, and clinical effectiveness. Delays between the initial clinical assessment and treatment initiation may contribute to disease progression, increased treatment complexity, reduced patient adherence, and deterioration in oral health-related quality of life. Consequently, time-to-treatment (TTT) has emerged as an important performance indicator for evaluating the efficiency of healthcare delivery systems and identifying organizational barriers that may compromise access to care [20,21,22,23].

Within university dental clinics, treatment timelines are influenced by factors that extend beyond clinical urgency. Patient scheduling frequently depends on student availability, faculty supervision, academic calendars, laboratory workflows, equipment allocation, and coordination among multiple clinical departments. Although these educational requirements are intrinsic to academic institutions, they may unintentionally increase waiting times, particularly for complex multidisciplinary treatments involving sequential referrals across several specialties.

The increasing complexity of comprehensive dental rehabilitation further emphasizes the importance of monitoring treatment intervals. Contemporary management of patients requiring implant-supported rehabilitation, advanced periodontal therapy, oral surgery, or extensive prosthodontic treatment commonly involves multiple diagnostic and therapeutic stages performed by different clinical teams. Delays occurring at any stage of these coordinated pathways may accumulate over time, affecting both organizational efficiency and the overall patient experience.

Previous investigations have primarily evaluated waiting times in relation to emergency dental care, specialist consultations, or access to public oral healthcare services. However, considerably less attention has been devoted to treatment intervals within university dental hospitals, where organizational complexity differs substantially from conventional healthcare environments. Moreover, few studies have examined treatment timing together with referral dynamics and continuity of care as complementary dimensions of healthcare quality. Simultaneously evaluating these organizational indicators may provide a more comprehensive understanding of patient flow and identify opportunities for improving multidisciplinary clinical pathways.

### 1.5. Network Analysis as a Tool for Evaluating Healthcare Organization

Network science has been used in healthcare to describe relationships among clinicians, departments, and organizations [24,25,26,27,28,29,30]. In the present analysis, nodes are dental services and edges are consecutive service attendances by the same anonymized patient code. This deliberately limited definition makes the model reproducible from routine data while avoiding unsupported claims about referrals or clinical coordination.

The analysis uses four main concepts. Degree counts the number of distinct services connected to a service; weighted transition counts measure the volume of observed transitions; PageRank summarizes the relative prominence of receiving transitions from other prominent services; and betweenness measures how often a service lies on shortest network paths. Density describes how many of the possible directed service pairs were observed, and modularity assesses the strength of a proposed grouping of services.

Patient-sharing and professional networks have been studied in hospital medicine, primary care, oncology, and integrated care systems [12,13,14,15,24,25,26,27,28,29,30]. Their interpretation depends on how an edge is defined. Unlike networks based on documented referrals or clinician relationships, the present model uses temporal adjacency of service attendance and should be interpreted as an exploratory map of institutional activity.

The intended audience is academic dental leadership, clinical-service managers, dental informatics teams, and researchers using routine administrative data. The immediate outcome is a reproducible description of activity and patient transitions. The model is not an implementation-ready decision system and does not estimate treatment delay, satisfaction, or clinical benefit; those outcomes require prospectively defined variables and validation.

### 1.6. Routinely Collected Data, Reproducibility, and Reporting Standards

Routinely collected health data can support efficient service evaluation, but their secondary use requires transparent reporting of source populations, linkage, cleaning, coding, missingness, validation, and analytical provenance. RECORD extends STROBE specifically for studies based on administrative and electronic healthcare data [31].

Data-quality assessment should address completeness, conformance and plausibility, because apparently precise administrative counts may represent different clinical constructs across services [32,33]. Patient linkage and deduplication require particular care when identifiers, dates and service labels originate from heterogeneous exports [34].

Dental education frameworks emphasize integrated clinical competence and coordinated patient management, but curricular organization can also shape where and when patients are treated. These educational and organizational effects should therefore be considered when interpreting service-level activity and patient-sharing networks [35,36,37].

### 1.7. Study Objectives and Analytical Boundaries

The study aimed to (1) reconcile institutional activity from 1 January 2025 to 30 June 2026; (2) define and describe service activity, patient denominators, and the institutional first-visit ratio; (3) reconstruct multiservice trajectories as consecutive service attendance rather than formal referral; and (4) calculate reproducible network measures that can help academic dental leaders identify patterns for subsequent operational review. The analysis was descriptive and was not intended to demonstrate improvements in workflow, waiting time, satisfaction, or clinical outcomes.

## 2. Materials and Methods

### 2.1. Study Design, Setting and Reporting Period

This was a retrospective ecological and administrative-data study of 18 clinical services in a university dental clinic in Spain. The reporting period included all 2025 data represented in the supplied sources and the available 2026 data through 30 June 2026. The study followed STROBE principles [5] and the RECORD extension for studies based on routinely collected health data [31]. The service was the ecological unit for descriptive comparisons; anonymized patient codes were used only for deduplication and longitudinal ordering.

### 2.2. Source Hierarchy and Single Source of Truth

Three source layers were distinguished. First, the official institutional report supplied the definitive descriptive totals and service-level counts. Second, the mother workbook supplied treatment codes and anonymized patient codes for audit and deduplication. Third, the multiservice chronology supplied ordered patient stages for transition reconstruction. Descriptive totals were not replaced by ad hoc counts from the mother workbook. Conversely, network statistics were not taken from narrative claims or service aggregates; they were recalculated from the individual chronology.

### 2.3. Temporal Restriction and Data-Quality Rules

All individual trajectory analyses used an inclusive cutoff of 30 June 2026. Dates later than this cutoff were excluded. The mother workbook contained an exact duplicate sheet (Hoja4 and Hoja5); the duplicate was removed for patient-level audits. The research team received only anonymized records and had no access to names or other direct patient identifiers. Any research extract retained or shared must preserve this anonymized status.

### 2.4. Derivation of Treatment Activity

The official total of 98,685 treatment records was derived by summing the treatment-record count across the 18 institutional services. Independently, grouping the official detail by treatment code produced 476 distinct codes whose frequencies also summed to 98,685. This two-way reconciliation—service total and code total—was used as an arithmetic integrity check. A treatment record is an administrative coded row and does not, by itself, prove completion, clinical success or a unique procedure episode. Multiple codes may belong to one appointment, and one patient may contribute records in several services.

For each treatment code, its count and percentage of all treatment records were calculated as n/98,685. Treatment codes were associated with their corresponding clinical service or specialty; however, in the absence of a validated institutional codebook, individual codes were not interpreted as representing specific clinical procedures.

### 2.5. Appointments, First Visits, Patients and ICI

Unique appointments and first visits followed the definitions used in the official report. The institutional continuity indicator (ICI) was calculated as first visits divided by unique appointments for each service and for the institution (1351/24,327). The ratio describes the recorded mix of first visits relative to appointment volume under local registration practices. It is useful for auditing differences in service activity and documentation, but it is not a validated measure of longitudinal continuity, care quality, or comparative service performance. Values are especially sensitive to whether a service registers encounters as first visits. Service-level patient counts describe distinct patients within each service; their sum (13,026) is non-additive because one patient may appear in several services. Deduplication of anonymized patient codes yielded 7260 unique institutional patients.

### 2.6. Construction of the Patient-Transition Matrix

For each patient, valid dates were sorted chronologically. Services recorded on the same date were grouped into a joint stage to avoid an arbitrary within-day order. For each pair of consecutive stages, directed edges were generated from every service in the earlier stage to every different service in the later stage; self-edges were excluded and repeated occurrences increased edge weight. For example, if services A and B occurred in one dated stage and services C and D in the next, the algorithm generated four directed transitions: A→C, A→D, B→C, and B→D. Thus, one pair of multiservice stages can contribute more than one edge. This produced an 18 × 18 weighted directed adjacency matrix. A return event was defined as re-entry to a service after at least one intervening stage without that service.

### 2.7. Network Statistics and Reproducibility

**Figure 1:**
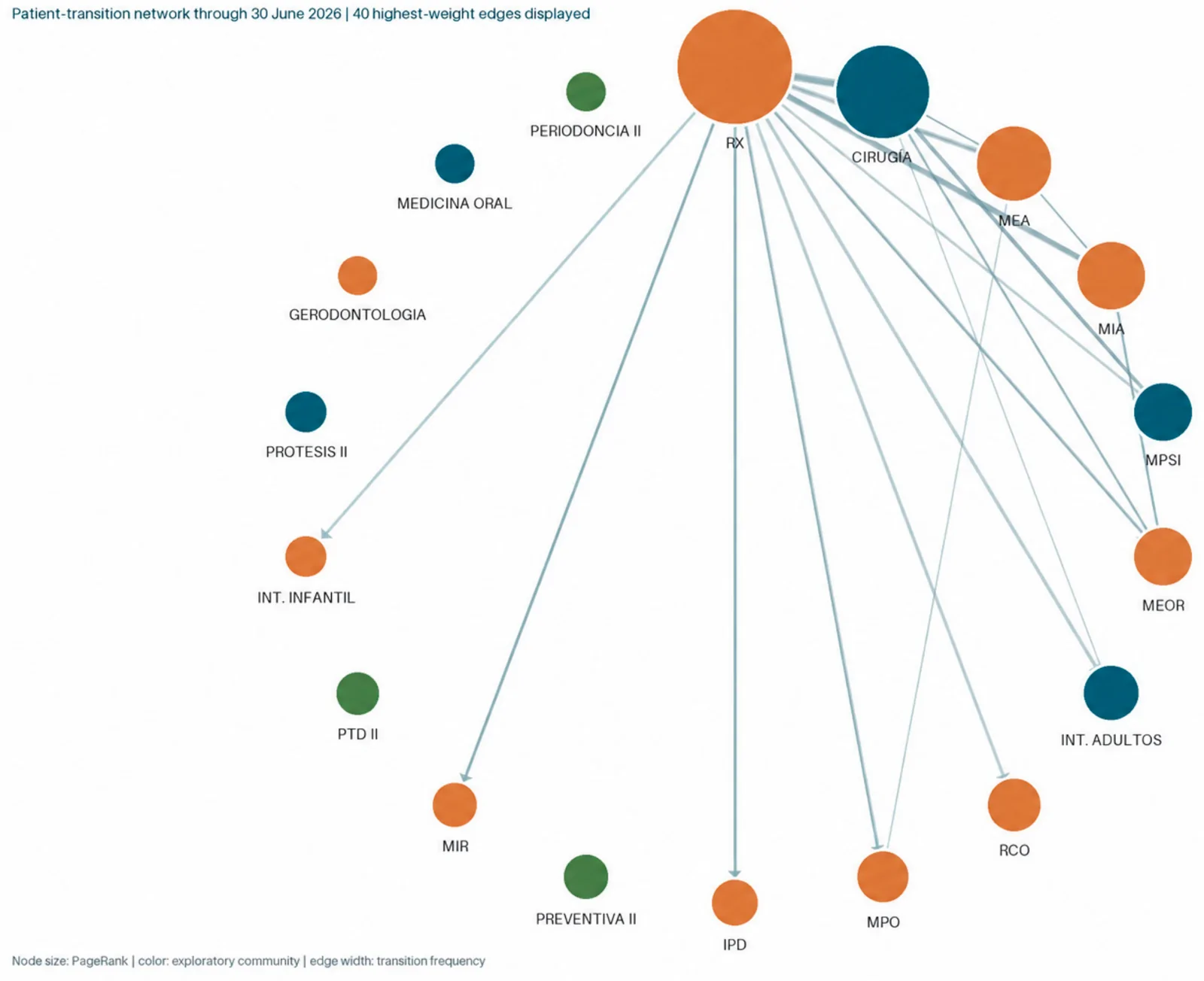
Patient-transition network from 1 January 2025 to 30 June 2026. All 18 services are displayed as nodes. Node size is proportional to PageRank and edge width represents transition frequency. Only the 40 highest-weight directed edges are shown for legibility; all 253 directed pairs were retained in metric calculations, so an absent visible edge does not indicate an absent transition. Node colors show an exploratory algorithmic partition only. Because modularity was very low (Q = 0.042), the colors must not be interpreted as clinically validated groups. The visualization represents consecutive service attendance, not documented patient-transition pathways.

In-degree and out-degree counted distinct incoming and outgoing service pairs. Weighted PageRank used transition frequencies and damping 0.85 [2]. Directed betweenness was calculated on the unweighted graph using the Brandes algorithm and normalized over possible ordered node pairs [3]. Modularity was calculated on the weighted undirected projection using a deterministic greedy merge procedure [4]. Because community solutions can depend on algorithm and resolution, modularity and community membership were treated as exploratory. Density equaled observed directed pairs divided by 18 × 17. Figure 1 displays only the 40 largest edges for legibility; all 253 edges were retained in the calculations.

### 2.8. Analyses Considered but Not Estimable

Kaplan–Meier estimation requires a defined time origin, event time or censoring time for each individual [38]. Cox regression additionally requires a valid risk set, covariates and assessment of proportional hazards [39,40]. The supplied files did not consistently identify the first eligible consultation, first therapeutic intervention, episode boundaries, censoring, competing events or referral orders. Therefore Kaplan–Meier curves, log-rank tests, Cox coefficients, hazard ratios, confidence intervals and Schoenfeld diagnostics were not calculated or retained.

### 2.9. Statistical Presentation

Counts, percentages and network metrics are descriptive. No causal effect is estimated. Spearman correlations generated during exploratory audit were not retained as primary results because only 18 ecological service observations were available and the ICI has heterogeneous registration meaning across services.

### 2.10. Detailed Treatment-Record Audit Trail

The derivation was audited in two independent directions. In the service-first route, the 18 service counts were summed up and compared with the institutional total. In the code-first route, every non-empty administrative treatment code was grouped across services, producing 476 distinct codes; their 98,685 records were then reconciled to the same institutional total. Equality of both routes was required before results were accepted.

### 2.11. Handling of Multiple Codes and Repeated Attendance

No attempt was made to collapse multiple codes occurring for the same patient, service or date into a single clinical procedure, because a validated episode-building rule was unavailable. This decision preserves the official treatment-record definition. Appointment counts were treated separately from treatment-record counts, preventing multiple coded items within one appointment from artificially increasing the appointment denominator. Patient histories were normalized as identifiers for deduplication but were not exposed in outputs.

### 2.12. First-Visit Classification and Denominator Control

The first visit variable was inherited from the previously reviewed service files. The conservative classifier identified encounters that necessarily represented an initial assessment for oral surgery, implants or extractions when those services were assessed. The manuscript uses only the official aggregate of 1351 first visits. Because first-visit registration was absent in some services, including RX, zero values were retained and not imputed.

### 2.13. Network Reconstruction Audit

Reconstruction was performed patient by patient. The quality-control sequence comprised date parsing, exclusion of records after 30 June 2026, service-name harmonization, same-day grouping, chronological ordering, construction of consecutive-stage edges, exclusion of self-edges, aggregation of repeated edges and independent verification that the final node set contained the same 18 services as the official report. The reported density, degree, PageRank, betweenness and modularity all derive from this frozen matrix.

### 2.14. Sensitivity and Interpretive Safeguards

The temporal correction from a provisional end date in December to 30 June 2026 changed the individual network to 253 directed pairs and 8777 weighted transitions. Metrics from the earlier broader window were discarded. No numerical result was retained solely because it appeared in a previous manuscript. Where raw administrative fields could support more than one clinical interpretation, the least inferential label was selected—for example, patient transition rather than referral and treatment record rather than completed procedure.

## 3. Results

### 3.1. Reconciled Institutional Activity

The official report contained 98,685 treatment records, 24,327 unique appointments and 1351 first visits. The global ICI was 5.55%. Table 1 presents the full service-level derivation. Cirugía Bucal and MIA together contributed 37,682 records (38.2% of the institutional total). RX had the largest appointment count (6021) but no registered first visits. This contrast illustrates that the ICI primarily reflects local first-visit registration and activity mix and must not be interpreted as a ranking of continuity or quality.

### 3.2. Treatment-Code Derivation

The treatment details contained 476 distinct codes. Their frequencies summed to exactly 98,685, matching Table 1 with zero residual difference. Table 2 shows the 20 most frequent codes. The largest code was VAL (7303; 7.4%), followed by RXD-01 (4983; 5.0%) and REVCIR (4605; 4.7%). These labels are reported as administrative codes without unverified clinical expansion.

### 3.3. Cutoff-Restricted Multiservice Chronology

The institutional dataset contained 7260 unique patients. The patient-transition analysis was restricted to the 3947 patients who attended more than one service and could therefore contribute at least one between-service transition; patients attending only one service are represented in institutional totals but not in the network. After the 30 June 2026 cutoff, the multiservice group contributed 16,347 dated stages. Of these patients, 1235 (31.3%) returned to at least one previously visited service, generating 2502 return events. The network therefore describes multiservice trajectories rather than all patient pathways in the clinic.

### 3.4. Reproducible Network Results

The cutoff-restricted matrix contained all 18 services, 253 of 306 possible directed pairs, and 8777 weighted transitions, giving a high density of 0.827. Because most service pairs were connected, differences in binary connectivity were limited and alternative short paths compressed betweenness values. Table 3 reports all service-level metrics. PageRank was highest for RX (0.202) and Oral Surgery (0.149), but these values indicate structural prominence in the observed transition data, not greater organizational importance, care quality, or a coordinating role. Frequent imaging within dental care is a plausible contributor to the RX result. The greedy partition returned three groups, but modularity was only Q = 0.042 and did not support strong clinical compartmentalization.

Figure 1 is an intentionally truncated visualization: it displays only the 40 highest-weight transitions for readability, whereas all 253 directed service pairs were retained in the calculations. Consequently, the absence of a visible edge in the figure does not mean that no transition occurred. Node size represents PageRank, node color represents the exploratory partition, and edge width represents transition frequency. The figure describes patient transitions and not documented referrals.

### 3.5. Unsupported Time-to-Event Outcomes

No Kaplan–Meier estimate, Cox model or hazard ratio met the minimum data-definition requirements. Consequently, no survival figure, regression table, *p*-value or proportional-hazards statement is included. Any future analysis must begin with a frozen patient-level episode table containing index consultation, treatment initiation, censoring date, event status and prespecified covariates. Specifically, the first-visit code (1VIS) was not recorded consistently across all services (RX reported zero first visits), and the date of first therapeutic intervention could not be systematically identified because the available codes did not reliably distinguish diagnostic, consultative, administrative, and therapeutic procedures.

### 3.6. Distribution and Concentration of Institutional Activity

The activity distribution was markedly heterogeneous. Cirugía Bucal contributed 19,108 records (19.4%) and MIA 18,574 (18.8%); together they represented 37,682 records, or 38.2% of the official total. MPO, MEOR and RX added 10,963, 9267 and 8061 records, respectively. At the opposite end of the distribution, Medicina Oral, Periodoncia II, Preventiva II, PTD II and Prótesis II each contributed fewer than 500 records. These differences describe coding volume and should not be interpreted as comparative productivity without adjustment for code structure, clinical complexity, staffing and teaching schedules.

### 3.7. Appointment Burden, First Visits and Patient Denominators

RX accounted for 6021 unique appointments, the largest service-specific appointment count, while reporting no first visits. Cirugía Bucal contributed 4161 appointments and 329 first visits, giving an ICI of 7.9%. IPD had the highest service ratio (100/451; 22.2%), whereas several teaching and diagnostic services had low or zero values. The 13,026 service-patient sum exceeded the 7260 institutional histories by 5766 counts because the former intentionally repeats patients across services.

### 3.8. Treatment-Code Concentration

The three leading administrative codes—VAL, RXD-01 and REVCIR—accounted for 16,891 records (17.1%). The 20 most frequent codes are shown in Table 2. The distribution of 476 treatment codes reflects substantial heterogeneity in administrative coding. While codes could be linked to their corresponding clinical service, interpretation at the level of specific clinical procedures or broader treatment categories would require an institutionally validated codebook and expert review.

### 3.9. Recurrent Multiservice Trajectories

Within the corrected temporal window, 1235 of 3947 multiservice patients (31.3%) returned to at least one previously attended service after an intervening stage, generating 2502 return events. The services most frequently re-entered were RX (780 return events among 604 patients), Cirugía Bucal (475 events among 334 patients), and MIA (338 events among 249 patients), followed by MPSI (154), MEA (141), and MEOR (127). These counts were recalculated using the same patient-stage algorithm as the network analysis and differ from the provisional values suggested during review. Without reason-for-return codes, they remain descriptive and cannot distinguish planned review, staged care, complications, diagnostic re-evaluation, or a new episode.

## 4. Discussion

### 4.1. Principal Findings

This revision separates two evidentiary layers. Institutional activity is anchored to the official report and reconciles both by service and by all 476 treatment codes. Network results come from a new patient-level analysis covering data through 30 June 2026. This prevents descriptive totals from being mixed with provisional individual filters and prevents advanced metrics from being copied from unsupported narrative claims.

### 4.2. Interpretation of Service Activity and ICI

The large difference between treatment records and appointments shows that a scheduled encounter can contain multiple coded items. Treatment-record volume should therefore not be equated with completed procedures or patients treated. The ICI is a local first-visit-to-appointment ratio intended to describe registration and activity mix. It is not the Bice–Boxerman continuity index [1], and comparisons such as 0% for RX versus 22.2% for IPD may mainly reflect service-specific registration practices. The ICI cannot rank continuity or quality without a validated and consistently applied first-visit definition.

### 4.3. Interpretation of Network Metrics

RX had the highest PageRank, plausibly because imaging commonly occurs before or between dental services. PageRank therefore identifies RX as structurally prominent within the observed sequences, not as more important, higher quality, or responsible for coordinating referrals. The high network density also limits discrimination based on connectivity and compresses betweenness. Low modularity indicates weak separation under the selected algorithm; named clinical communities would exceed the evidence.

### 4.4. Strengths

Strengths include an explicit source hierarchy, reconciliation of all treatment codes, institutional patient deduplication, an exact temporal cutoff, same-day stage handling, a fully specified transition rule and removal of unsupported survival outputs. Table 1, Table 2 and Table 3 and Figure 1 are cited in the text.

#### 4.4.1. Comparison with Healthcare Patient-Sharing Networks

Patient-sharing networks have identified professional communities and organizational patterns in large healthcare systems [12,13,14,15]. The present study differs because its nodes are dental services rather than individual clinicians, and its edges represent consecutive attendance rather than professional collaboration or documented referral. Its significance for academic dentistry lies in demonstrating that routinely collected data can be reconciled and transformed into an auditable map of multiservice activity. The model is suitable for descriptive screening and hypothesis generation, but it is not yet adequate as an implementation or performance-management tool. Application across dental electronic health records would require standardized service labels, documented referral reasons, episode boundaries, consistent first-visit coding, waiting-time variables, and linkage to patient-reported and clinical outcomes.

#### 4.4.2. Academic-Clinic Context

University clinics operate simultaneously as healthcare providers and educational environments. Treatment sequencing may reflect case complexity, student competency requirements, faculty availability, laboratory stages and academic calendars. Consequently, high transition volume can represent appropriate multidisciplinary care, educational workflow or administrative fragmentation; the present data cannot separate these mechanisms.

#### 4.4.3. Complete Treatment Derivation

The arithmetic reconciliation of 98,685 records across 18 services and 476 treatment codes is a central strength. It demonstrates internal consistency of the official descriptive report. Nevertheless, record counts are not synonymous with completed clinical procedures: several codes may be attached to one encounter, and administrative items, reviews, radiographs or planned stages may coexist with definitive treatment codes.

#### 4.4.4. Interpretation of PageRank and Betweenness

PageRank emphasizes services receiving transitions from other influential services [2], whereas betweenness depends on the shortest-path structure [3]. In a network with a density of 0.827, alternative short paths are abundant, so normalized betweenness values become compressed. RX therefore may achieve a high PageRank through routine diagnostic sequencing without functioning as a referral coordinator.

#### 4.4.5. Interpretation of Modularity

Modularity evaluates whether within-group edge weight exceeds that expected under a null configuration [4,26,27]. The observed Q of 0.042 indicates weak separation under the prespecified greedy procedure. Community colors in Figure 1 are therefore exploratory visual aids and must not be treated as validated clinical divisions.

#### 4.4.6. Why Survival Modeling Remains Excluded

Kaplan–Meier and Cox methods require patient-level event definitions and censoring information [38,39,40]. A sequence of coded appointments cannot establish when a patient first became eligible for treatment or when therapeutic intervention began. Reporting survival curves or hazard ratios from these files would create false precision; this exclusion is a substantive correction, not a missing analysis.

### 4.5. Limitations

The study is retrospective, single-center, and dependent on administrative coding. Its services include institution-specific postgraduate programs and teaching units, so the observed topology is likely shaped by local curricula, staffing, scheduling, coding, and access arrangements. The numerical centrality values, transition frequencies, and community assignment should not be generalized directly to university dental clinics organized differently. External validity requires replication using harmonized definitions across institutions. The official descriptive report and individual workbooks are not identical analytical objects. Treatment status and clinical completion were not uniformly established. The chronology includes only multiservice patients, does not prove referrals, and omits single-service pathways from the transition network. No validated clinical codebook, urgency, diagnosis, severity, episode boundary, treatment-start endpoint, or referral order was available.

### 4.6. Reproducibility Requirements for Future Analysis

- Freeze a de-identified patient-level dataset with a documented extraction date and cutoff.
- Subject to explicit institutional authorization, release the aggregated 18 × 18 weighted transition matrix and analysis code used for PageRank, betweenness, and modularity. The matrix is not included with the present revision because the current data-use authorization does not permit public dissemination; it remains available on reasonable request through the corresponding author, subject to institutional approval.
- Prespecify same-day transitions, self-edges, edge weights, PageRank damping and community algorithm.
- For survival analysis, the required variables are: (1) index date, defined as the first documented clinical assessment at which the patient became eligible for treatment; (2) event date, defined as the first qualifying therapeutic procedure; (3) censoring date, defined as the last known clinical contact or 30 June 2026; (4) event status, coded 1 for treatment initiation and 0 for censoring; and (5) prespecified covariates including service, patient age, treatment complexity, urgency classification, and documented referral status. None of these variables were consistently available in the supplied data.
- Report Kaplan–Meier denominators at risk and Cox coefficients, hazard ratios, 95% confidence intervals and proportional-hazards diagnostics only after validation.

### 4.7. Implications for Clinical Management

Despite the inability to confirm formal referrals or estimate treatment delays, the results provide an operational screening framework for academic dental leadership. The 2502 return events and the concentration of activity around Oral Surgery, MIA, and RX identify patterns that can guide targeted audits of scheduling capacity, diagnostic sequencing, and multidisciplinary documentation. They do not demonstrate inefficiency or improved outcomes. A practical next step is to validate selected high-volume pathways with clinicians and then link standardized referral, waiting-time, completion, satisfaction, and outcome variables prospectively. Only after that validation should the model be used to design or evaluate workflow changes.

## 5. Conclusions

The institutional report covering 1 January 2025 to 30 June 2026 contained 98,685 treatment records, 24,327 unique appointments, and 1351 first visits. Deduplication identified 7260 unique patients, while the transition network represented the 3947 patients with multiservice trajectories. The non-additive total of 13,026 describes service-specific patient relationships rather than unique people.

The multiservice chronology supported an exploratory network of 8777 transitions and 253 directed service pairs. The analysis provides academic dental leaders with an auditable way to identify high-volume service connections for targeted operational review, but it neither documents formal referrals nor demonstrates improved workflow or patient outcomes. Future implementation requires standardized electronic health record fields for referral reasons, episode boundaries, treatment timing, completion, satisfaction, and clinical outcomes, followed by external validation across differently organized dental institutions.

## Figures and Tables

**Table 1:** Official institutional activity by service through June 2026.

Service	Treatment Records	Share	Unique Appointments	First Visits	ICI	Service Patients
Oral Surgery (Cirugía Bucal)	19,108	19.4%	4161	329	7.9%	1610
MIA (Advanced Implant Dentistry)	18,574	18.8%	3091	180	5.8%	1010
MPO (Periodontology and Osseointegration)	10,963	11.1%	1427	102	7.1%	747
MEOR (Endodontics and Restorative Dentistry)	9267	9.4%	1103	107	9.7%	507
RX (Radiology)	8061	8.2%	6021	0	0.0%	4757
MPSI (Implant Prosthodontics)	6182	6.3%	1795	111	6.2%	558
MEA (Advanced Endodontics)	5232	5.3%	1570	120	7.6%	1001
MIR (Implant Dentistry and Rehabilitation)	4463	4.5%	417	47	11.3%	152
Integrated Adult Dentistry	4324	4.4%	1031	103	10.0%	530
Integrated Pediatric Dentistry	4126	4.2%	1253	30	2.4%	738
RCO (Clinical Dentistry Residency)	3283	3.3%	896	77	8.6%	559
IPD (Implant Dentistry for Daily Practice)	2300	2.3%	451	100	22.2%	214
Gerodontology	1223	1.2%	228	21	9.2%	117
Prosthodontics II	471	0.5%	261	14	5.4%	57
Preventive Dentistry II	413	0.4%	253	3	1.2%	208
PTD II (Dental Pathology and Therapeutics II)	413	0.4%	198	7	3.5%	137
Periodontology II	160	0.2%	71	0	0.0%	54
Oral Medicine	122	0.1%	100	0	0.0%	70
TOTAL/SUM	98,685	100.0%	24,327	1351	5.6%	13,026 *

* Service patients are distinct anonymized patients counted separately within each service. Because the same patient can attend more than one service, the sum of 13,026 is non-additive and answers how many service-specific patient relationships were observed. Deduplication across the institution yielded 7260 unique patients.

**Table 2:** Twenty most frequent treatment codes in the official report.

Rank	Treatment Code	Records	Share of 98,685
1	VAL	7303	7.40%
2	RXD-01	4983	5.05%
3	REVCIR	4605	4.67%
4	1VIS	3796	3.85%
5	QUITAR	3480	3.53%
6	IMP54	3045	3.09%
7	IMP49	2786	2.82%
8	IMP52	2760	2.80%
9	IMP20AA	2712	2.75%
10	IMP51	2675	2.71%
11	IMP20A	2620	2.65%
12	CRG-01	2421	2.45%
13	IMP58A	2111	2.14%
14	IMP58B	2079	2.11%
15	IMP58C	2078	2.11%
16	IMP58D	2059	2.09%
17	PERIO-09	1520	1.54%
18	REVPROT	1463	1.48%
19	IMP20	1396	1.41%
20	IMP05	1214	1.23%

**Table 3:** Recalculated network metrics from individual trajectories through 30 June 2026.

Service	In-Degree	Out-Degree	Weighted Incoming Transitions	Weighted Outgoing Transitions	Betweenness	PageRank	Exploratory Community
RX (Radiology)	17	17	1585	3685	0.025	0.202	2
Oral Surgery (Cirugía Bucal)	17	17	1480	1250	0.025	0.149	1
MEA (Advanced Endodontics)	17	17	1029	634	0.025	0.101	2
MIA (Advanced Implant Dentistry)	16	17	990	716	0.023	0.088	2
MPSI (Implant Prosthodontics)	15	15	515	374	0.007	0.062	1
MEOR (Endodontics and Restorative Dentistry)	17	17	532	384	0.025	0.059	2
Integrated Adult Dentistry	17	16	459	345	0.016	0.054	1
RCO (Clinical Dentistry Residency)	15	16	463	316	0.010	0.049	2
MPO (Periodontology and Osseointegration)	15	14	431	278	0.008	0.045	2
IPD (Implant Dentistry for Daily Practice)	13	15	293	183	0.006	0.032	2
Preventive Dentistry II	16	12	132	81	0.007	0.027	3
MIR (Implant Dentistry and Rehabilitation)	13	13	267	182	0.003	0.027	2
PTD II (Dental Pathology and Therapeutics II)	13	13	136	79	0.003	0.025	3
Integrated Pediatric Dentistry	5	8	197	78	0.000	0.020	2
Prosthodontics II	13	11	89	64	0.004	0.018	1
Gerodontology	11	12	72	50	0.001	0.015	2
Oral Medicine	13	12	57	37	0.004	0.014	1
Periodontology II	10	11	50	41	0.002	0.013	3

Note: In-degree and out-degree count distinct connected service pairs; weighted incoming and outgoing transitions count all observed transitions. Community membership is exploratory because modularity was very low (Q = 0.042).

## Data Availability

The data presented in this study are available on reasonable request from the corresponding author. The research team received only fully anonymized administrative records and had no access to direct patient identifiers. The patient-level data and aggregated transition matrix are not publicly available because their reuse and dissemination remain subject to institutional authorization. The 18 × 18 weighted matrix, data dictionary, and analysis code may be shared subject to approval by Universidad Rey Juan Carlos.

## Abbreviations

ICI	Institutional Continuity Indicator
STROBE	Strengthening the Reporting of Observational Studies in Epidemiology
RECORD	REporting of studies Conducted using Observational Routinely collected health Data
RX	Radiology service
HR	Hazard ratio
CI	Confidence interval
MIA	Advanced Implant Dentistry Master’s Program
MEA	Advanced Endodontics Master’s Program
MPSI	Implant Prosthodontics Master’s Program
MEOR	Endodontics and Restorative Dentistry Master’s Program
MPO	Periodontology and Osseointegration Master’s Program
MIR	Implant Dentistry and Rehabilitation Master’s Program
RCO	Clinical Dentistry Residency
IPD	Implant Dentistry for Daily Practice Expert Program
PTD II	Dental Pathology and Therapeutics II
